# 2-step deep learning model for landmarks localization in spine radiographs

**DOI:** 10.1038/s41598-021-89102-w

**Published:** 2021-05-04

**Authors:** Andrea Cina, Tito Bassani, Matteo Panico, Andrea Luca, Youssef Masharawi, Marco Brayda-Bruno, Fabio Galbusera

**Affiliations:** 1 IRCCS Istituto Ortopedico Galeazzi, Via Riccardo Galeazzi 4, 20161 Milan, Italy; 2 Department of Spine Surgery III, IRCCS Istituto Ortopedico Galeazzi, Via Riccardo Galeazzi 4, 20161 Milan, Italy; 3 Department of Physiotherapy, Sackler Faculty of Medicine, The Stanley Steyer School of Health Professions, Tel Aviv University, Tel Aviv, Israel

**Keywords:** Health care, Medical research, Engineering

## Abstract

In this work we propose to use Deep Learning to automatically calculate the coordinates of the vertebral corners in sagittal x-rays images of the thoracolumbar spine and, from those landmarks, to calculate relevant radiological parameters such as L1–L5 and L1–S1 lordosis and sacral slope. For this purpose, we used 10,193 images annotated with the landmarks coordinates as the ground truth. We realized a model that consists of 2 steps. In step 1, we trained 2 Convolutional Neural Networks to identify each vertebra in the image and calculate the landmarks coordinates respectively. In step 2, we refined the localization using cropped images of a single vertebra as input to another convolutional neural network and we used geometrical transformations to map the corners to the original image. For the localization tasks, we used a differentiable spatial to numerical transform (DSNT) as the top layer. We evaluated the model both qualitatively and quantitatively on a set of 195 test images. The median localization errors relative to the vertebrae dimensions were 1.98% and 1.68% for x and y coordinates respectively. All the predicted angles were highly correlated with the ground truth, despite non-negligible absolute median errors of 1.84°, 2.43° and 1.98° for L1–L5, L1–S1 and SS respectively. Our model is able to calculate with good accuracy the coordinates of the vertebral corners and has a large potential for improving the reliability and repeatability of measurements in clinical tasks.

## Introduction

Nowadays we are facing an exponential increase in the availability of biomedical data that open different challenges and opportunities in healthcare research^1^. Among this abundance of data, we have large sets of medical images (CT, MRI or radiographs) that can be used for various tasks such as the detection or the classification of pathologies or the localization of relevant anatomical structures and points of interest. Regarding spine imaging, landmarks localization has indeed a high relevance in the clinical perspective because it allows characterizing spine alignment in term of angles, distances and shape that could be useful e.g. for surgical planning or monitoring the progression of deformities^2^. Such parameters are usually measured manually with rule and protractor on the printed images or with the help of dedicated software on digital images, leading to a potential impact on accuracy and reproducibility due to intra- and inter-observer variabilities^3^. To overcome these issues, in the last decades there has been an increase of the development of computer-aided diagnosis systems (CAD) that can reduce errors and increase the efficiency of image analysis^4^, although in most cases still require manual intervention. The use of fully-automated software tools could permit overcoming these limitations and has the potential for impacting both medical research and clinical practice. Recent studies^5,6^ have demonstrated the feasibility of the development of tools to automatically measure spine parameters, thanks to the spread of novel deep learning techniques and the use of high computational power provided by graphics processing units (GPUs).

In fact, in recent years artificial intelligence and in particular deep learning have been used increasingly in medical imaging, thanks to their state-of-the-art performances, superior in many cases to expert human observers. In^7,8^ the authors developed an automatic tool for the localization of vertebrae using computed tomography (CT) images. The research groups identified vertebral centroids with good accuracy but did not propose relevant applications to be applied in the clinical practice. In a work by Jacobsen et al.^9^, the authors used deep learning for the automatic segmentation of cervical vertebrae. The results showed that the errors in identifying the positions of the vertebral corners were not negligible. Moreover, the images were limited to the cervical region and the dataset was rather small, making this approach impractical in ordinary clinical settings. Other models were developed with the specific purpose of calculating spine angles such as the sacral slope (SS), the pelvic tilt (PT), the pelvic incidence (PI) in the sagittal plane and the Cobb angle in the coronal plane^10,11^. In^10^, the calculation of the angles was performed using only specific points detected by the deep learning model, implying that the model can be used only for specific angles. In^11^, a model that calculates the position of the vertebral corners overcoming the limitations of^10^ was developed. However, in both studies all the radiographs presented a standardized field of view, which avoided the problem of understanding which vertebrae were visible in each specific image. Another work by Galbusera et al.^12^ aimed at automatically calculating spine angles using biplanar images acquired by EOS system (EOS Imaging, Paris, France). Also in this case the images were standardized (same field of view) and the calculation of the angles showed room for improvements. Other works focused on the 3D reconstruction of the spine using automatic^13,14^ or semi-automatic models^15^. In^13,14^ the authors applied a statistical model and a Convolutional Neural Network respectively to accurately reconstruct the spine shape and evaluated the model accuracies calculating the Euclidean distances in mm between the model predictions and the ground truth. In^15^ a manual intervention was required before the model could calculate the relevant parameters.

In this work, we propose a deep learning approach for the automatic identification of the coordinates of the vertebral corners in sagittal x-rays images of the lumbar spine, without a priori knowledge about vertebral level. These landmarks could be used to calculate relevant radiological parameters such as segmental and global lordosis, as well as spinopelvic parameters such as sacral slope. Moreover, the model can process sagittal radiographs of the lumbar spine with different resolutions and different fields of view. Although our model was not trained specifically on images that contain spinal instrumentation we found that it could also process those images with good visual performances.

## Results

In this study, we developed a model that use deep learning to identify and localize the vertebrae on sagittal x-ray images. In particular, we used a 2-step model (see Material and Methods for details): in step I, we applied 2 Convolutional Neural Networks (CNN 1 and CNN 2) to label the vertebrae present in the image (CNN 1) and to calculate the x and y coordinates of the 4 corners (CNN 2) on the whole image. The 4 corners were used to crop the single vertebrae and to apply another CNN (CNN 3) on single vertebra images to refine the localization of step I and then to calculate the coordinates of the corners on the original image (step II).

The localization of the landmarks predicted by our model was visually good. The model resulted suitable for processing radiographs presenting different resolutions, different fields of view, as well as images of patients with spinal instrumentation. It should be noted that we had only a few images with spinal instrumentation both in the training set and in the test set, but the model was able to localize the vertebrae also in such images.

**Figure 1:**
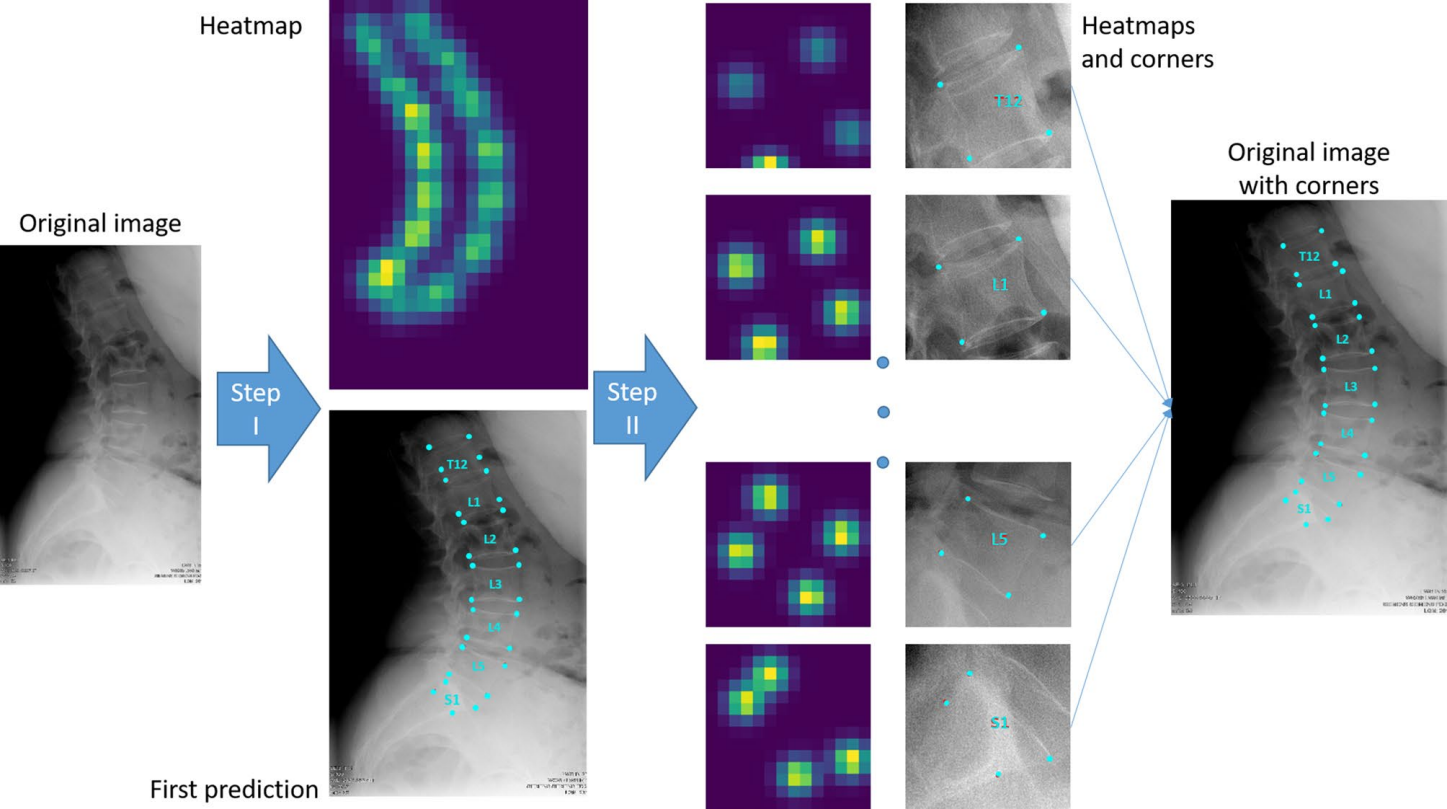
Predictions of the 2-step model for a representative image. The image from the test set is input into the model and processed to obtain an image fully annotated with the corners and names of the vertebrae. The heatmaps were generated using the Matplotlib library (https://matplotlib.org/) in Python 3.8 (https://www.python.org/downloads/release/python-380/).

**Figure 2:**
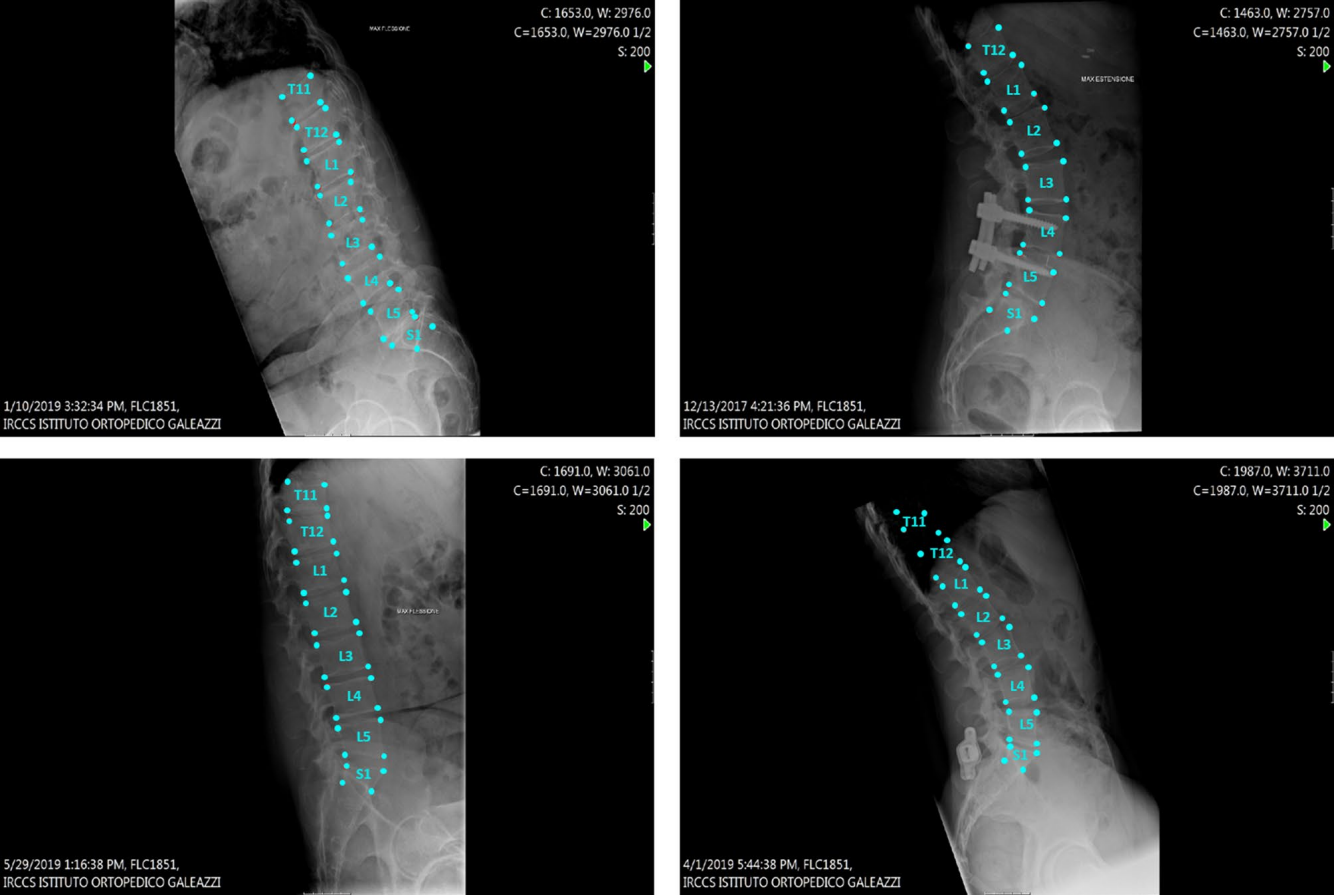
Four random images from the test set. These images have large black edges that do not affect the model performances.

**Figure 3:**
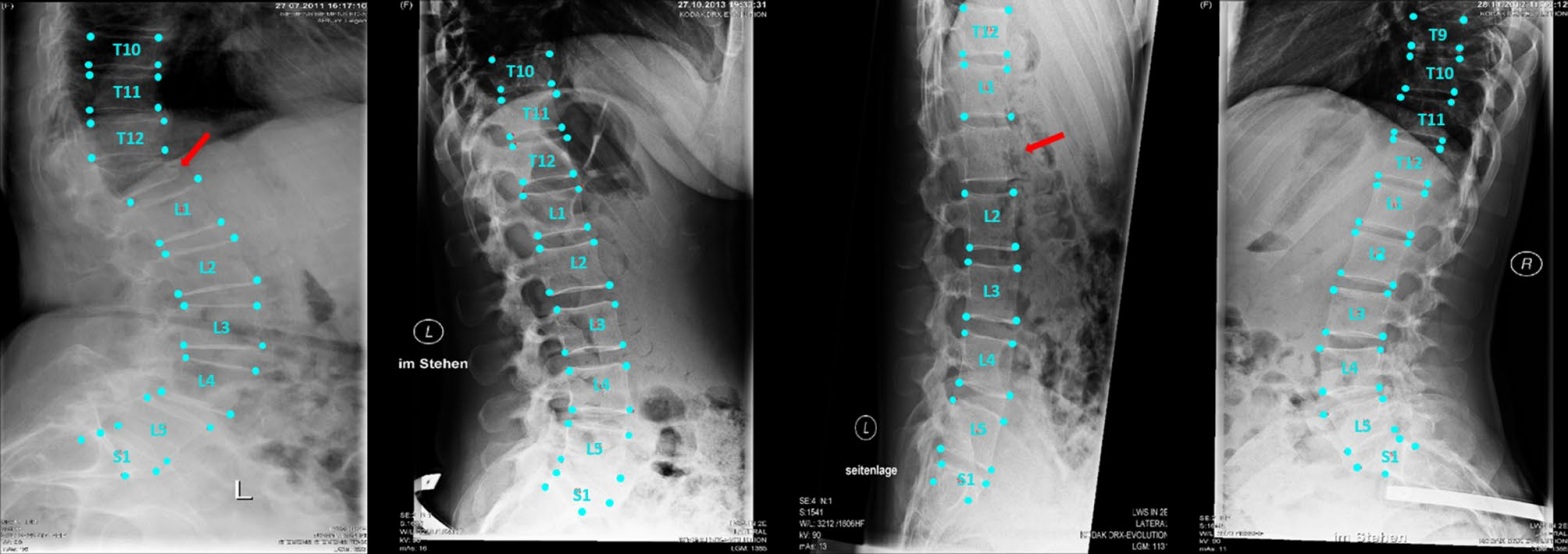
Four random images from the test set. The red arrows indicate the vertebrae missed by the model. In the first image, a fractured vertebra was missed. In the third image from the left the step I model (vertebra identification) missed the T11 vertebra and the error was propagated to step II.

**Figure 4:**
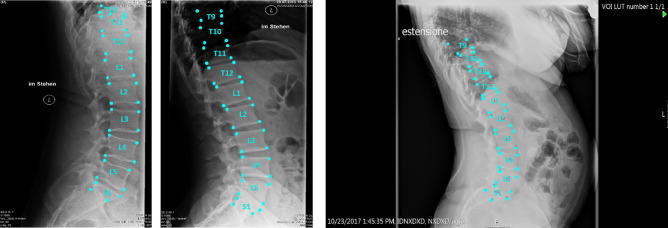
Examples of images hard to understand also for a human observer. In the first image, the model does not find the correct coordinates of T10 since the vertebra is cropped. In the second image, the top part is black making distinguishing a vertebra impossible. In the third image, the brightness is too high to localize correctly the vertebrae at the top.

An example of the step by step model for landmarks prediction on a representative radiograph is reported in Fig. 1: in step I (CNN 1 + CNN 2), the original image is processed to obtain a heatmap representation of the localization of the first landmarks for all the visible vertebrae. The obtained coordinates are then used, in step II, for cropping the single vertebra and obtaining the corresponding representation as heatmap, thus allowing refining landmarks prediction. Finally, the position of the vertebral corners in the original image is calculated by means of geometrical transformations. The advantage of such an approach can be verified by observing the progressive refinement in the identification of the position of vertebral corners, which although acceptable in many cases by step I (see vertebrae from L2 to S1 in Fig. 1) improves evidently after applying the subsequent step 2 (see L1 and T12). Therefore, quantitatively, we can observe an improvement of the weighted mean of the median errors from 2.20 to 1.98% and from 2.04 to 1.68% for x and y coordinates respectively (see Table 1). The model performed well both when processing images with the region of interest in the foreground and with cases with black edges filling large portions of the image (Fig. 2). In some cases, the model missed the identification of some vertebrae or cannot recognize their correct position (Figs. 2, 3, 4). However, it is worth noting that in many of these cases, understanding where the vertebrae are located would be difficult even for an expert human observer (Fig. 4).

**Table 1:** Errors calculated for the single vertebrae.

Vertebra	Median error x after step I (%)	Median error x after step II (%)	Median error y after step I (%)	Median error y after step II (%)	Number of samples
T9	3.94	2.95	8.89	5.64	15
T10	2.86	2.50	3.47	2.96	66
T11	2.56	2.50	3.09	2.50	139
T12	2.52	2.32	2.53	2.06	188
L1	2.16	1.97	2.11	1.84	194
L2	1.71	1.56	2.12	1.71	195
L3	1.52	1.59	1.79	1.44	195
L4	1.59	1.52	1.68	1.40	195
L5	2.01	1.86	2.21	2.05	195
S1	3.27	2.44	2.51	2.38	195
Weighted mean	2.20	1.98	2.04	1.68	–

The first 2 columns report the median of the absolute error related to the x and y vertebra dimension (Eqs. 1 and 2). The last row sums up the results to have an overall evaluation (Eqs. 5 and 6).

**Figure 5:**
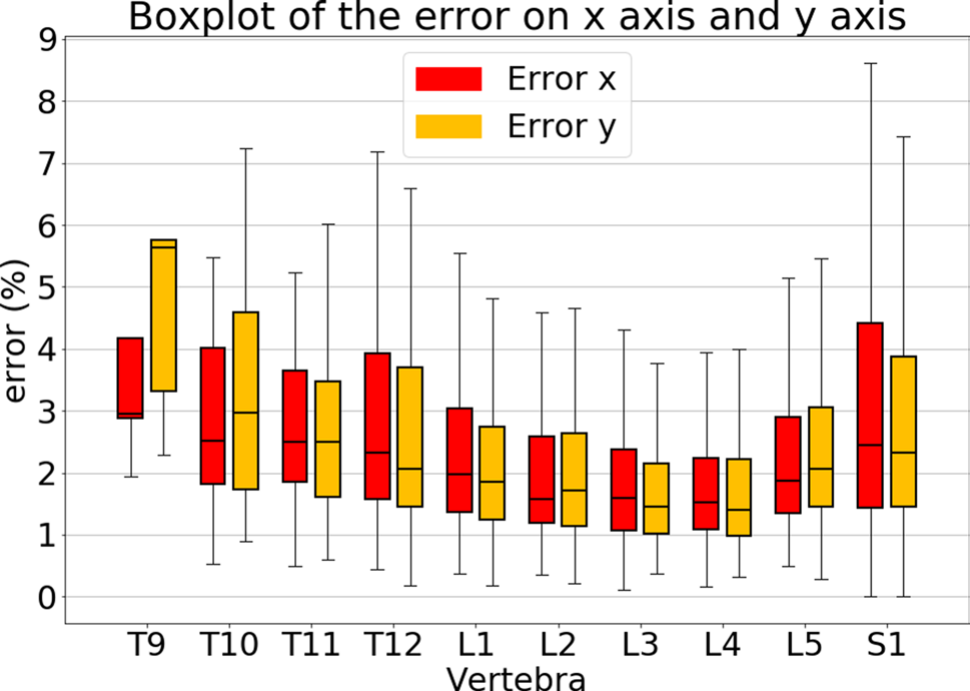
Boxplot of the absolute errors for x (in red) and y (in orange) axis. The errors are in percentage and are relative to the x and y dimension of the vertebra (width and height). (Eqs. 1 and 2).

The quantitative analysis confirmed the performance of our 2-step model in the vertebral corners localization. The accuracy in detecting vertebrae was 98.4%. A vertebra is correctly detected when the CNN 1 label it correctly as L1, L2 etc. so the accuracy is computed as the ratio between the correctly labeled vertebrae and the total number of vertebrae. The analysis of the absolute error between the ground truth and the predictions revealed that, in general, the model was equally accurate in the localization on both the x-axis and the y-axis (Fig. 5). The medians of the absolute errors, except for T9, are below 4% of the vertebral width indicating good performance both for x and y. It should be noted that T9 and T10 were underrepresented in the training set, thus explaining the slightly worse performance in correspondence of these vertebrae*.* For S1, the medians of the x and y errors were 2.44% and 2.38% of the vertebral width, respectively. The weighted means of the median errors (Eqs. (5, 6)) were 1.98% and 1.68% for x and y respectively (Table 1). The maximum errors in the estimation were 77% and 120% for x and y respectively. These errors resulted from images in which the model missed a vertebra and so the error between the ground truth and the prediction was necessarily high.

**Figure 6:**
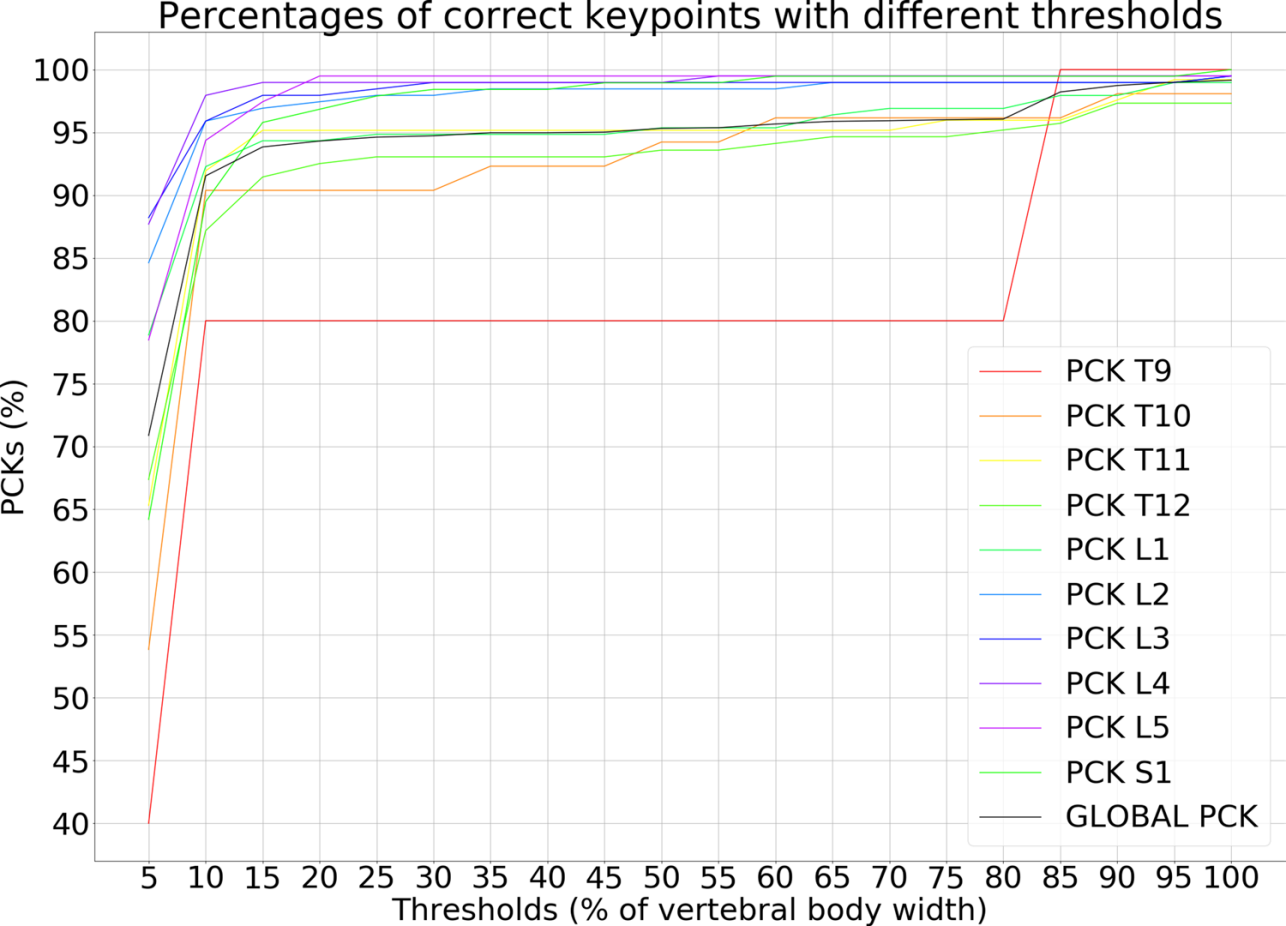
Curves that shows how the PCKs change according to different thresholds of the horizontal length of the vertebrae.

As regards the Percentage of Correct Keypoints (PCKs), the analysis showed that using 10% of the width of the vertebral body as the threshold, the parameter resulted in 80% for T9, 87% for T12, 89% for S1, 90% for T10, 92% for T11 and L1, 94% for L5, 96% for L2 and L3, and 97% for L4. Moreover, the best performance was observed for the lumbar vertebrae, with a mean PCKs over all the thresholds of 95%, 97.6%, 98.1%, 98.6% and 98% for L1, L2, L3, L4, L5 respectively (Fig. 6). The calculation of L1-L5, L1-S1 and SS angle showed median absolute errors of 1.84°, 2.43° and 1.98°, respectively. The max absolute errors were 50°, 49° and 34° for L1-L5, L1-S1 and SS respectively. Moreover, the percentages of absolute errors above 5° were 15%, 20% and 16% for L1-L5, L1-S1 an SS. We chose 5° as the threshold because it is the error of human observers according to^16^.

**Figure 7:**
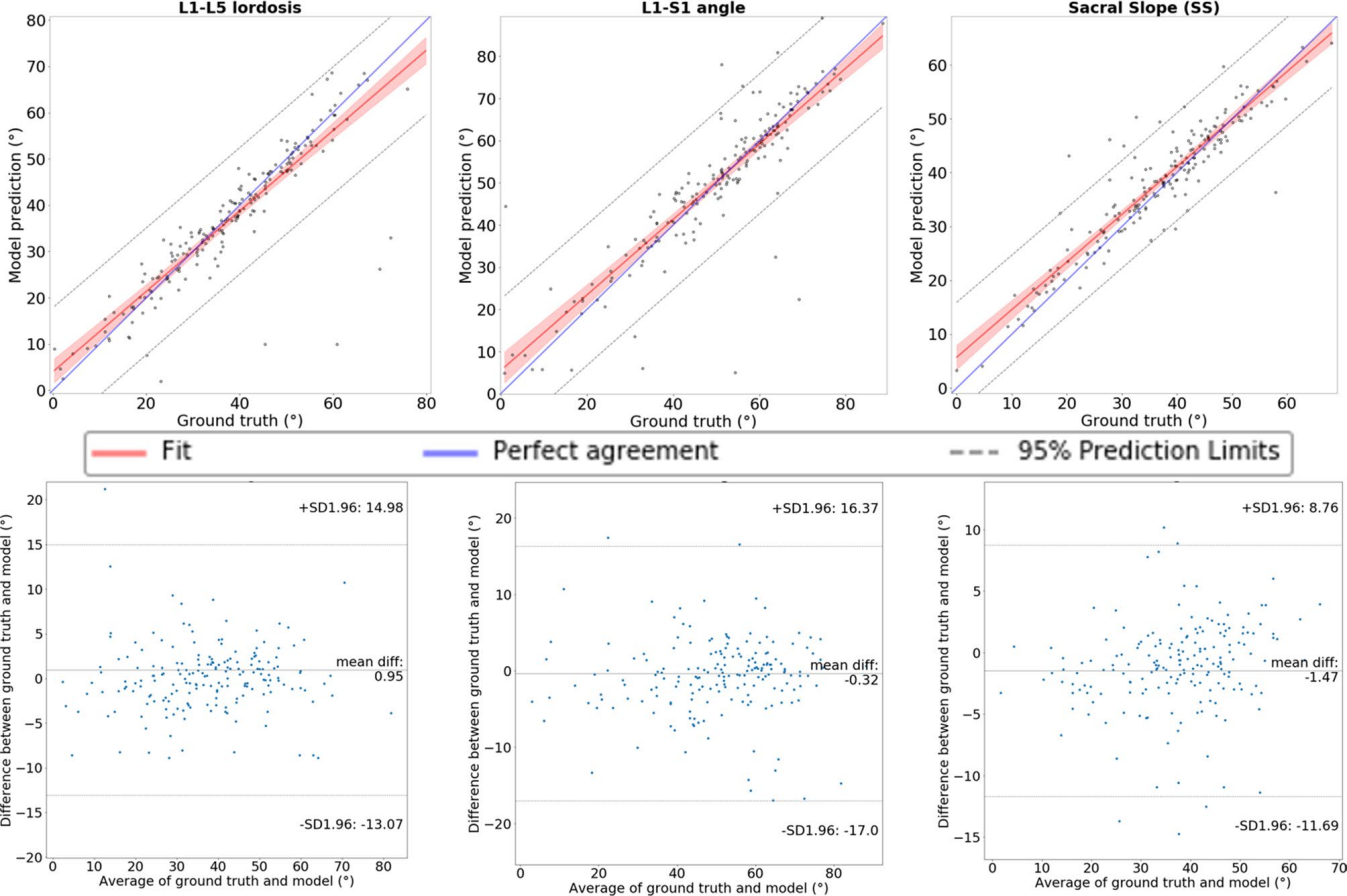
Regression analysis plots comparing the values of the ground truth angles and of the angles predicted by the 2-step model. From left to right: L1–L5 lordosis, L1–S1 angle and Sacral Slope (SS). The 95% confidence interval (dashed line), the 95% confidence limits of the prediction line (rendered in solid light red), the fit line (red line) and the perfect agreement (45° blue line) are shown. We can see that the fit is very close to the perfect agreement. The Bland–Altman graphs show a low mean difference between the ground-truth and the model prediction despite a non-negligible variability.

Regression analysis showed a high correlation between the ground truth and model prediction (Fig. 7). The high correlation is confirmed by the *R*^2^ coefficient in Table 2, although with not negligible values about the standard deviations (between 4.31° and 7.15°).

**Table 2:** Regression analysis comparing the angles calculated from the manually annotated vertebral landmarks (ground truth) and those calculated from the corners predicted by our model.

Angles	*R*^2^	SD
L1–L5	0.77	6.30°
L1–S1	0.75	7.15°
SS	0.81	4.31°

## Discussion

This paper presents a 2-step model for the fully automated identification and landmarks localization of thoracolumbar vertebrae in sagittal x-ray images. The approach has demonstrated high accuracy in identifying vertebrae and landmarks in radiographs with different resolutions and fields of view. In this regard, the identified coordinates allowed calculating clinically relevant parameters such as lordosis angles and sacral slope.

The qualitative analysis of the landmarks prediction showed a good visual performance: Figs. 2 and 3 confirmed the robustness of the model with a large variety of situations (noise, reduced field of view, spinal instrumentation and spine rotation in flexion–extension). For example, a qualitative comparison with previous data such as^9^ clearly highlights the better accuracy of our automatic annotation with respect to the state-of-the-art (Figs. 2, 3). However, the quantitative evaluation revealed that the differences between the model prediction and the ground truth could affect the implementation of such a method in the clinical practice.

Compared to previous works^10,12^ better results were achieved in the calculation of L1–L5 and SS angle. In particular, for L1–L5 we obtained *R*^2^ equal to 0.77 (standard deviation = 6.30°) between the ground truth and the model predictions, versus 0.62 (11.5°) reported in^12^. Concerning SS, we reported a mean absolute difference between ground truth and predicted values of 3.27° (standard deviation = 4.31°) versus 5° (3.4°) and 8.6° (8.5°) obtained by Korez et al*.*^10^ and Galbusera et al*.*^12^ respectively, as well as improved *R*^2^, 0.81, compared to 0.58 in^12^ and to 0.73 in^10^. However, the maximum errors in the angles calculation were too high (50°, 49° and 34° for L1–L5, L1–S1 and SS respectively). We did not implement a manual intervention to help the model to correct these large errors so the model cannot be used for this purpose in a clinical application setting.

In a clinical perspective, the developed model has the potential for a relevant impact, especially in the field of spinal degeneration and deformities. In its current status, it allows calculating relevant radiological parameters (lordosis, kyphosis, segmental wedging, sacral slope, eliminating intra- and inter-observer variability^3^ and manual processing time. Future developments may potentially allow for the estimation of other sagittal parameters such as pelvic incidence, pelvic tilt, sagittal vertical axis. However, it should be noted that such extensions require a substantial amount of work, an enlargement of the training set, and further validations.

A possible application of our model is to provide a consistent way of automatically measuring the correction achieved after surgery for the treatment of spinal disorders such as spondylolisthesis. Moreover, the model could be used in the intra-operative phase; indeed, the achievement of the target when correcting spinal deformities is commonly assessed only post-operatively, whereas a real-time quantitative check of the correction during the surgery is currently not feasible, apart from approximate estimations by means of fluoroscopic imaging. In the research context, our approach can speed up the process of annotation to perform different tasks on x-ray images. For example, in order to compute the range of motion and center of rotation in flexion–extension paired radiographs, the manual annotation of all the radiographs is required before performing the computation. In this regard, the exploitation of the proposed model can allow for building an end-to-end pipeline that takes as input a pair of radiographs and provides as output the range of motion and the center of rotation of the motion segment. The developed model is also expected to provide an advantage to research projects in which accuracy and reproducibility of the measurements are fundamental, e.g. to improve the homogeneity of data collected in multicentric clinical trials or in the frame of trials conducted for regulatory purposes, for example for the marketing authorization of new spinal implants.

Despite the good performances generally superior to the state-of-the-art, we think that our model needs some improvements before clinical use. As a matter of fact, the model missed some vertebrae in the images (Fig. 3) leading to incorrect labeling and localization, potentially affecting the calculation of the angles. In several cases, the gross errors could be related to anatomical features which were underrepresented in the training dataset. For example, the presence of vertebral fracture, which is extremely rare in the training set, compromised the correct identification of the vertebra in Fig. 2. In this regard, we hypothesize that a targeted expansion of the training dataset would be effective in mitigating such limitation. The angles calculation using landmarks has room for improvement. The maximum errors are too high to make the model suitable for a real world application.

Moreover, we are aware that deep learning models need a large number of images to be trained effectively. For this reason, we decided to use Transfer Learning instead of building and training a model from scratch to obtain better performances^17^. In particular, we used model parameters guaranteeing state-of-the-art performances in the ImageNet challenge, and retrain only a few layers (see Methods section for details) on the new task. However, we think that using more images, adding images of cases that the model was not able to manage correctly (images with fracture for example) and retraining a model from scratch would potentially lead to an improvement in the results. Unfortunately, very large datasets annotated with landmarks coordinates are not currently available nor easy to create.

In conclusion, we presented a 2-step model for the automatic processing of sagittal x-ray images. The model has been proved to be able to identify the vertebrae in the image and the corresponding landmarks with a good precision. The identification of the landmarks allowed measuring clinically relevant parameters such as L1–L5 and L1–S1 lordosis angles and sacral slope with good accuracy. We think that the exploitation of deep learning approaches can provide automatic models with a huge impact on musculoskeletal radiology as well as on other medical fields.

## Methods

The deep learning model aims at providing an entire pipeline that takes as input a sagittal x-ray image of the lumbar spine and produces as output the (x, y) coordinates in pixels of the corners of the vertebrae visible in the image, and to identify each vertebra (e.g. ‘T12’, ‘L1’, etc.).

### Dataset

**Figure 8:**
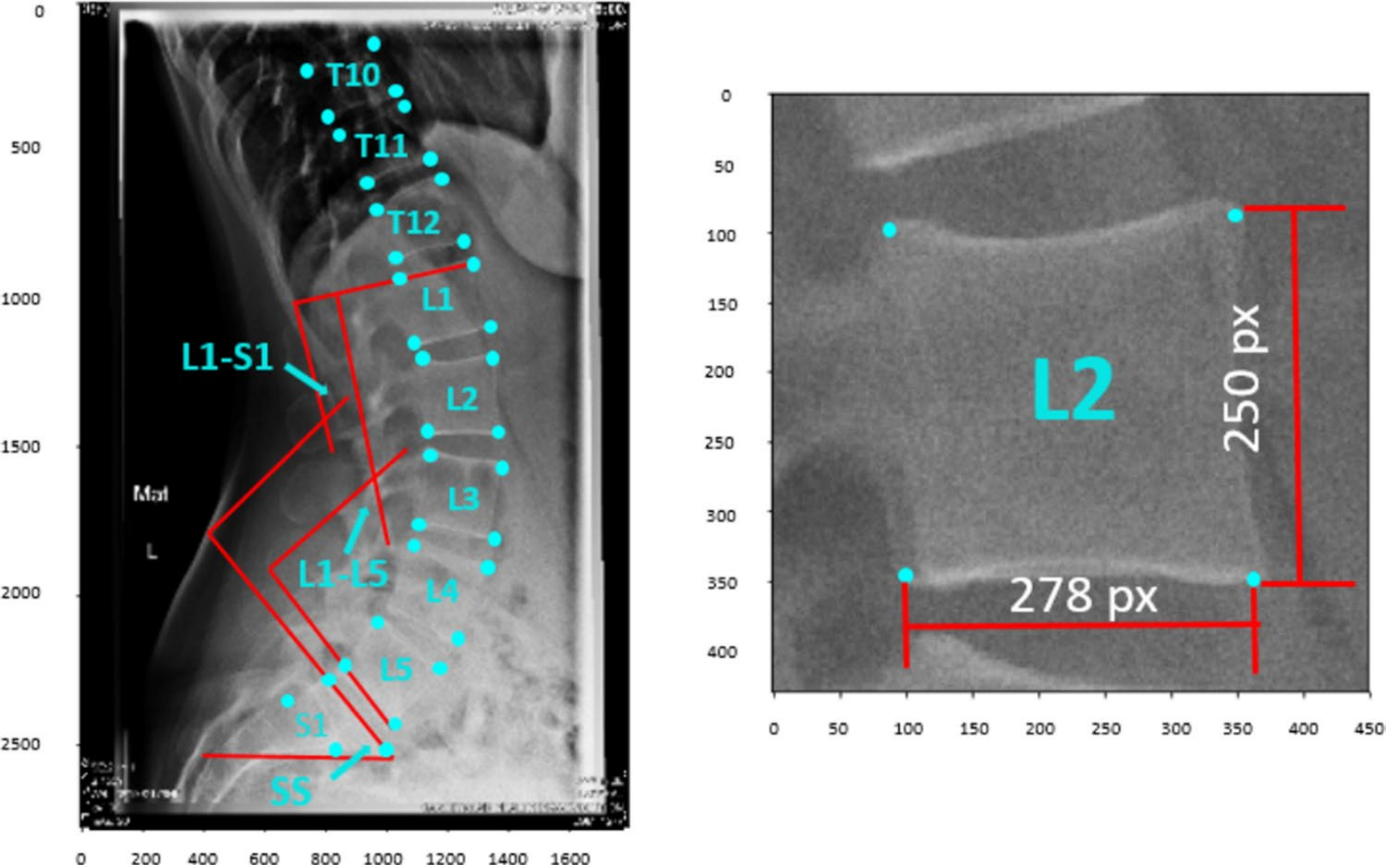
On the left an image of the spine with corners and lumbar angles. On the right a single vertebra with the x and y dimensions in pixel.

**Figure 9:**
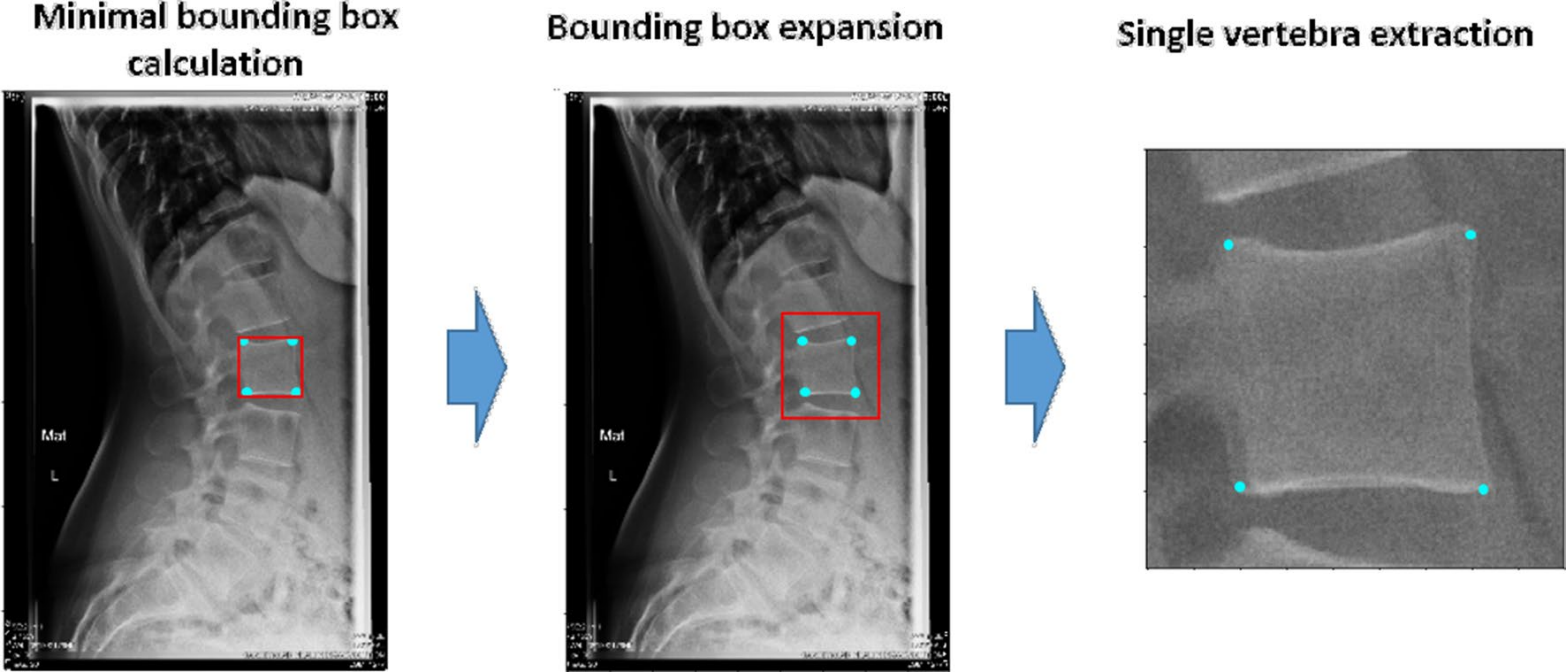
Example of the extraction of a single vertebra image for L2 vertebra. We used a process that will be applicable also during the testing phase or in the clinical practice without manual intervention.

The dataset consisted of 10,193 sagittal uncalibrated x-ray images of the lumbar spine acquired from the database of IRCCS Istituto Ortopedico Galeazzi (Milan, Italy) and fully anonymized before any use. All subjects gave informed consent for scientific and educational use of the images. We included all the images collected to have a relevant number of samples. Therefore, the dataset includes normal, pathological and instrumented images as well as images of young and elderly people. All images were manually labeled with the (x, y) coordinates of the vertebral corners (Fig. 8). The images were annotated with a purposely developed C++ application, in which the user marked the 4 corners of each vertebra visible in the image, and assigned a label to the vertebra corresponding to spine level from C2 to S1 (Fig. 9). Since the annotation process is a time-consuming task, all the images were annotated by only one person. To ensure that this limitation had a minor influence on the quality of the results, we calculated the inter and intra-observer variability on a subset of 30 images annotated by 4 human observers, by calculating the corresponding correlation coefficients as reported in^18^. The inter-observer correlation coefficients were 0.99 and 0.96 for x and y coordinates respectively while the intra-observer correlation coefficients were 0.99 for both x and y coordinates; these values indicate high consistency in the labeling process. Then, the dataset was split into three subsets of 10,140, 588, and 195 images, respectively, for training, validation, and test of the model. Moreover, we calculated the mean, the maximum and the minimum of the 3 angles that we considered in this study. The mean values were 36,3°, 49,9° and 35,8°, the maximum values were 86,8°, 89,9° and 89,4° and the minimum values were 0.1°, 0.1° and 0° for L1–L5, L1–S1 and SS respectively.

### 2-step model

**Figure 10:**
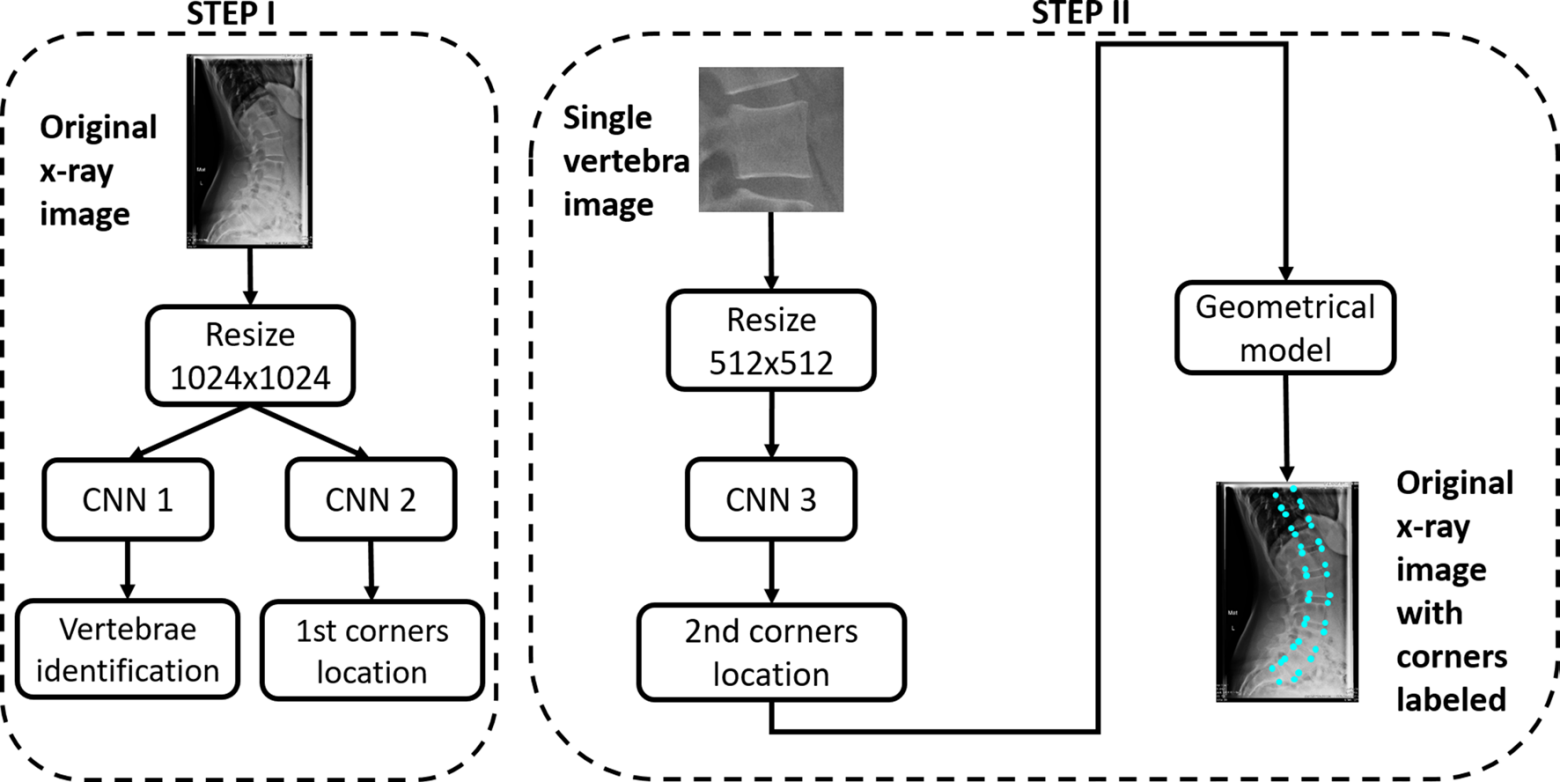
2-step model. The output of each step is the input of the next step. In a real-world setting, the user must only input the original x-ray image and the model will automatically calculate the coordinates of the corners.

Our approach consisted of 2 consecutive steps (Fig. 10). In step I, the original image was first resized to 1024 × 1024 pixels, and two Convolutional Neural Networks (CNNs) were used to identify the spine level of the visible vertebrae (CNN 1) and to calculate the coordinates of the vertebral corners (CNN 2). In step II, a cropped image was obtained for each identified vertebra, and another CNN (CNN 3) was applied to identify the coordinates of the corners after resizing the cropped image to 512 × 512 pixels. This strategy was chosen to provide higher accuracy in processing the vertebral corners by exploiting a smaller pixel size compared to the 1024 × 1024 image used in step I. Finally, we used simple geometrical transformations to map the corners calculated in step II to the original image. Regarding images resizing, we used the open-cv library (https://opencv.org/) with an inter-cubic filter; the dynamic range has not been modified and the original aspect ratio was not preserved.

*Step I* To enhance the multiclass classification task (presence/no-presence of vertebrae), we randomly cropped each image along the y-axis to allow the processing of radiographs with different fields of view. In fact, since the sacrum is visible in the majority of cases, this spine level could be categorically assigned to the most caudal visible vertebra in the identification process. To avoid such limitation, we randomly cropped the images in the lower part to have L5, L4 or L3 as the most caudal visible vertebra. In particular, the crop was made by generating a random y-coordinate that was in the lower half of the image and using this point as the cut point with the condition that at least 3 vertebrae remained visible. This procedure was performed only during the training process.

In addition, we performed data augmentation on the training set with random rotations and horizontal flipping. The images, after the resize, were normalized to have the pixels in the range [0, 1].

Concerning the model (CNN 1), we used a ResNet50 model^19^ pre-trained on ImageNet^20^ exploiting the Transfer Learning technique^21^. Hence, we decided to freeze the weights of the deeper layers until Bottleneck block 3 of the ResNet and retrain only the last part of the model. Moreover, we replaced the last layer of the original ResNet model with a fully connected layer with 24 neurons, one for each vertebra. The output of the network is a 24 elements vector assuming values of 1 or 0 indicating the presence or absence of the vertebra, respectively. Since the task is a multi-label binary classification, we used a sigmoid activation function for each output neuron minimizing the binary cross-entropy loss. In the training process, we monitored the loss value on the validation set and we saved iteratively the parameters of the network when the loss of the current epoch resulted lower than the best loss in the previous ones.

Regarding the training of CNN 2 for corners localization, after the first tests using only rotations and flipping as augmentation techniques, we decided to use also elastic transformations and noise addition to increase the robustness of the model. The augmentations were implemented using imgaug library (https://imgaug.readthedocs.io/en/latest/), which allows applying transformations both to images and coordinates. Also for CNN 2, we used Transfer Learning and froze the deeper layers to avoid overfitting in the training set. In particular, among the tested architectures, Inception V3^22^ was used since verified as providing the best performance since its auxiliary output improved the landmarks localization. Moreover, a differentiable spatial to numerical transform (DSNT) layer^23^, a convolutional fully differentiable layer which preserves the spatial generalization^12^, was implemented as top layer, due to its state-of-the-art performances for landmarks localization tasks. Therefore, we replaced the original Inception output layers (both the auxiliary and the main output) with a DSNT layer. Since up to 24 vertebrae can be potentially visible in the images, we used 24 DSNT layers, one for each vertebra, with 4 output channels (4 features maps), one for each corner (x, y) coordinates. The DSNT layers convert the 24 spatial heatmaps generated by the fully convolutional network (FCN) to numerical coordinates resulting in a 24 × 4 × 2 matrix. The output dimension means that we have 24 vertebrae, 4 vertebral corners for each vertebra and 2 coordinates for each corner (x, y). The model is trained to minimize the Euclidean distance between the predicted coordinates and the ground truth as suggested in the github repository (https://github.com/anibali/dsntnn) of the authors of^23^. Following the indication in the same repository, the ground truth coordinates have been normalized in the range [− 1, 1] for training.

*Step II* To obtain the cropped images to train CNN 3, we calculated the centroids and the minimal bounding box area (considering x and y maximum and minimum coordinates) for each vertebra, we extended the bounding box area by 70% and extracted the single vertebra image (Fig. 9). It should be noted that the coordinates of the original images were adjusted to match the new image reference system where the origin is placed in the (x, y) coordinates of the top left corner of the bounding box used to crop the image. We performed data augmentation on the training set using random rotations, flipping, elastic transformations and noise. For this task, we implemented the Inception V3 model with only one DSNT layer as output layer with the 4 output channels (4 features maps), one for each vertebral corner.

As in CNN 2 of step I, the DSNT layer converts the unique spatial heatmap to numerical coordinates providing a 1 × 4 × 2 matrix, and the model is trained to minimize the Euclidean distance between the predicted coordinates and the ground truth. The output matrix represents 1 vertebra with 4 corners and 2 coordinates x and y.

At this point, we found the corners coordinates in the 512 × 512 images and we applied the appropriate geometrical transformations to calculate the coordinates in the original reference system; we first resized the coordinates to match the dimensions of the single vertebra image and finally we translated the coordinates back to the reference system of the original image by adding the (x, y) coordinates of the top left corner of the bounding box used to crop the single vertebra (that is the origin of the reference system in the cropped image) (Fig. 9).

As regards model implementation, we used PyTorch^24^, a deep learning framework developed mainly for research purposes and written in Python. For training and validation, we used a Linux workstation equipped with a NVIDIA Titan Xp GPU. At the beginning of the study, we run each model for a few epochs (30 epochs) to find the best combination of learning rate and batch size. The best hyperparameter values for the multilabel classification (CNN 1) and the landmarks localization in step II (CNN 3) were a learning rate of 0.0001 and a batch size of 16 while for the landmarks localization model of step I (CNN 2) were a learning rate of 0.001 and a batch size of 8. For the final training, we run the model for 200 epochs, and we used a method that reduced the learning rate by a factor of 0.1 if the loss did not improve for 10 epochs in a row (ReduceLROnPlateau in PyTorch).

### Evaluation

We assessed the performance of the model both qualitatively and quantitatively. Whereas during the development of the 2-step model we evaluated the 3 CNNs individually, the metrics reported in the Results were calculated on the whole model on the original test set. Regarding the qualitative evaluation, a human observer checked if the vertebra and the corresponding coordinates of the corners were correctly placed on the images of the test set. Quantitatively, we computed the accuracy in the detection of the vertebrae by calculating the error between the predicted and the ground truth coordinates in the original images. For each vertebra, we computed the absolute errors, both for x and y coordinates, normalized by the width (Eq. (1)) and height (Eq. (2)) of the vertebral body, respectively. It should be noted that since S1 has only an upper endplate only the 2 points on the upper endplate are considered in the calculation. The errors for the x and y coordinates were calculated as follows:

1 $$e\left( x \right)_{i}^{k} = \frac{{\left| {\hat{p}\left( x \right)_{i}^{k} - p\left( x \right)_{i}^{k} } \right|}}{{length\left( x \right)_{i}^{k} }}100\%$$

2 $$e\left( y \right)_{i}^{k} = \frac{{\left| {\hat{p}\left( y \right)_{i}^{k} - p\left( y \right)_{i}^{k} } \right|}}{{length\left( y \right)_{i}^{k} }}100\%$$

being $\hat{p}$ the predicted point, *p* the actual point (ground truth), *i* the *i-*th image, *k* the *k-*th vertebral level and *length *(*x*) and *length *(*y*) the width and height of the vertebral body. After verifying the non-Gaussian distribution of the errors by means of the Shapiro–Wilk test, we calculated the median value of the normalized errors:

3 $$median\left( x \right)^{k} = median\left( {e\left( x \right)^{k} } \right)$$

4 $$median\left( y \right)^{k} = median\left( {e\left( y \right)^{k} } \right)$$

where $\mu e\left( x \right)\;{\text{and}}\;\mu e\left( y \right)$ indicate the median of the error along x and y, *e*(*x*) and *e*(*y*) are the mean absolute error distributions for x and y respectively and *k* is the vertebral level*.*

An additional parameter for evaluating landmarks localization task is the Percentage of Correct Keypoints (PCKs)^25,26^. In our study, we set different thresholds according to different percentages of vertebral body widths and we considered a landmark as correctly classified if the Euclidean distance between the predicted and the actual point resulted less than the specific threshold. We calculated different curves to analyze the changes of PCKs in dependence of the increasing percentage of the width of each vertebra as the threshold for the correct identification of a point. In particular, we varied the percentage of the horizontal length from 5 to 100% with an increment of 5% and we computed the PCKs for each threshold.

We also calculated a global median for x and y median errors:

5 $$median\left( x \right) = \frac{{\mathop \sum \nolimits_{k = 0}^{V} median\left( x \right)_{k} N_{k} }}{{\mathop \sum \nolimits_{k = 0}^{V} N_{k} }}$$

6 $$median\left( y \right) = \frac{{\mathop \sum \nolimits_{k = 0}^{V} median\left( y \right)_{k} N_{k} }}{{\mathop \sum \nolimits_{k = 0}^{V} N_{k} }}$$

being *V* the number of vertebral levels, *N* the number of samples for the vertebra *k*, *µe*(*x*) and *µe*(*y*) the medians of the error of vertebra *k*.

Finally, by using the corner coordinates, we evaluated the prediction of 3 relevant radiological angles: L1–L5 lordosis, L1–S1, and the sacral slope (SS, calculated as the slope of the S1 endplate with respect to the horizontal line). In particular, the angles were calculated by computing the line that passes through the 2 points of the lower and upper endplate of the corresponding vertebra. For example, for L1–L5 angle, we calculated the angular coefficients of the line that passes through the 2 upper points of L1 and the one that passes through the 2 lower points of L5 and from those coefficients we computed the angle between the 2 lines.
